# The Problem of the Elderly Primipara

**Published:** 1912-06-01

**Authors:** 


### MATERNITY AND MOTHERING.

(Inquiries and Correspondence are invited)

### The Problem of the Elderly Primipara.

It is an undeniable fact that people marry nowadays later in life than was formerly the usual custom. The tendency is best marked, no doubt, among the educated middle classes, for economic reasons; but it does also affect, in a lesser degree, both the aristocracy and the labouring class. It is also true that the tendency affects men more than women, since man is still in a majority of cases the bread-winner, notwithstanding the entry of women into so many spheres of industrial activity formerly closed to them. Allowing for these counter-balancing factors, it is still true that women marry and bear children in the main at a somewhat older age than they were wont to; and that the "elderly" primipara is more frequently met with in consequence.

As to what constitutes a woman "elderly" from the point of view of her behaviour in her first confinement opinions differ. Some authorities take the age of twenty-eight as the dividing line between youth and middle-age in this respect; and there is the justification for this view that about that time of life various changes are determined, as shown by statistics of male and female births, etc. (see The Hospital, Jan. 27, 1912, p. 437—"Some Problems of Birth"). But since the intention of classifying obstetric patients as elderly or otherwise is to indicate a difference in their clinical reactions when in labour, the sole criterion must be based upon clinical study; and clinical experience would indicate a much later age as that at which any given primipara may reasonably be labelled "elderly" in an obstetrical sense. Probably thirty-two to thirty-four may be safely taken as the boundary ages separating the two classes; though only broad lines, based on averages, can be laid down.

Let us take it, then, for the sake of argument, that there is some difference in the average course of a first confinement, depending on the age of the mother; second and subsequent labours are not under consideration at all, for they differ in many respects from the first which have various peculiarities all their own. According to several authorities the average duration of labour in an "elderly" primipara is greater by two or three or four hours than in those who are under that age. Various suggestions are propounded to explain this, though it cannot be said that any single hypothesis affords a valid explanation of the fact, if fact it be.

The result of these academical discussions in obstetrical literature is that a certain number of people, especially in the educated classes, have got the impression that a first confinement in a middle-aged woman (obstetrically speaking) has trials and dangers decidedly greater than for one under thirty; and doctors are often consulted by expecting mothers who have the most exaggerated fears in this respect. The real truth probably is that a difference does exist, but that it is very slight; and further that in the average normal healthy patient it is practically negligible, for it is chiefly due to the fact that a certain small percentage of women between thirty and forty-five develop various conditions internally, which are not so common between twenty and thirty. So the woman of over thirty-three, let us say, in whom a medical examination reveals no defect or disease, is for all intents and purposes as likely to come successfully through the ordeal of her first maternity as is the woman ten or fifteen years her junior.

A communication on this subject of the "elderly" primipara by Dr. Kate Spain was read recently before one of the United States medical societies. This doctor holds that the factor of greatest importance in causing the differences above alluded to is the lesser muscular power (on the average) of the uterus as age advances. This she ascribes partly to the natural result of age, partly to the greater liability in the fourth decade to tumours and other extraneous influences which may affect the uterine muscle. She attaches almost no importance at all to the supposed greater rigidity of the cervix and perineum in the elderly, both of which have been regarded by various authorities as contributory causes. Nor does she believe that the greater size of the average child born of a mother turned thirty has much to do with the prolongation of labour; and she produces figures which appear to show that this difference in size is very slight indeed. Dr. Spain admits the well-known fact that elderly mothers bear a large excess of boys over girls; and it is well established that boys are larger and more troublesome in births than girls. Here is a factor which may prolong labour in the middle-aged.

At the end of her excellent review of the subject, however, she launches a suggestion which goes far to destroy the favourable impression formed of her clinical wisdom. This is nothing less than that labour should be induced four weeks before full term in the "elderly" primipara. This is intended, as the context shows, to apply to all cases, not merely to those with pelvic contraction or other demonstrable abnormality likely to interfere with labour. The suggestion requires most emphatic condemnation on two grounds. First, it is an obstetrical operation the slight risk of which cannot be said not to exist at all, and is not to be undertaken without a more valid reason. It carries risks of sepsis which even in the most advantageous circumstances can never be completely eliminated. Secondly, it means the birth of a child whose chances of survival and of vigour are nowhere near those of a child born at full term; and, inasmuch as the mother is, *ex hypothesi*, not young, it is all the more important that such offspring as she may chance to bear shall have the fullest possible chance of survival. For dispelling the bogeys which oppress some prospective mothers, Dr. Kate Spain deserves gratitude and thanks; but her programme of the induction of premature labour is meddlesome, and therefore bad, midwifery practice which will, we trust, find few adherents.

